# Advanced machine learning algorithms to evaluate the effects of the raw ingredients on flowability and compressive strength of ultra-high-performance concrete

**DOI:** 10.1371/journal.pone.0278161

**Published:** 2022-12-22

**Authors:** Yunfeng Qian, Muhammad Sufian, Oussama Accouche, Marc Azab

**Affiliations:** 1 School of Civil Engineering, Changsha University of Science & Technology, Changsha, PR China; 2 School of Civil Engineering, Southeast University, Nanjing, PR China; 3 College of Engineering and Technology, American University of the Middle East, Egaila, Kuwait; University of Sulaimani, IRAQ

## Abstract

The estimation of concrete characteristics through artificial intelligence techniques is come out to be an effective way in the construction sector in terms of time and cost conservation. The manufacturing of Ultra-High-Performance Concrete (UHPC) is based on combining numerous ingredients, resulting in a very complex composite in fresh and hardened form. The more ingredients, along with more possible combinations, properties and relative mix proportioning, results in difficult prediction of UHPC behavior. The main aim of this research is the development of Machine Learning (ML) models to predict UHPC flowability and compressive strength. Accordingly, sophisticated and effective artificial intelligence approaches are employed in the current study. For this purpose, an individual ML model named Decision Tree (DT) and ensembled ML algorithms called Bootstrap Aggregating (BA) and Gradient Boosting (GB) are applied. Statistical analyses like; Determination Coefficient (R^2^), Root Mean Square Error (RMSE), and Mean Absolute Error (MAE) are also employed to evaluate algorithms’ performance. It is concluded that the GB approach appropriately forecasts the UHPC flowability and compressive strength. The higher R^2^ value, i.e., 0.94 and 0.95 for compressive and flowability, respectively, of the DT technique and lesser error values, have higher precision than other considered algorithms with lower R^2^ values. SHAP analysis reveals that limestone powder content and curing time have the highest SHAP values for UHPC flowability and compressive strength, respectively. The outcomes of this research study would benefit the scholars of the construction industry to quickly and effectively determine the flowability and compressive strength of UHPC.

## 1. Introduction

A relatively newer type of concrete, i.e., Ultra-High-Performance Concrete (UHPC), is recognized due to its superior durability and significantly higher compressive strength [1–4]. The structures exposed to severe climatic/environmental stresses can specifically be benefitted from UHPC’s superior durability. The other advantages of incorporating UHPC comprise the concrete quantum reduction for a structure that will conserve the net space, ultimately reducing the construction time, equipment and labor required for the erection of precast structural members [1, 2]. However, more contents of fine particles, i.e., cement, silica fume, quartz powder, and quartz sand, are used in UHPC to achieve the high packing density in coarse aggregates, ultimately enhancing its cost but offering a positive environmental impact at the same time [5–10]. Thus, considering these drawbacks, which limit its broader application, industrial end-products like fluid catalytic cracking residue, recycled glass powder, and other Supplementary Cementitious Materials (SCM) like limestone powder are utilized as a partial replacement for silica fume, quartz powder, and cement [11]. Furthermore, incorporating fibers in concrete also results in enhanced mechanical properties [12–17]. In the literature, the UHPC behavior was also analyzed by incorporating silica fume, fluid catalytic cracking residue, and fly ash. The potential of cement and silica-fume as partial cement replacement was reported [18]. Accordingly, the database in the case of UHPC comprises contents for cement, fly-ash, silica-fume, slag, nano-silica, quartz powder, limestone powder, sand, coarse aggregates, super-plasticizers, fibers and water, type, strength-class and strength of cement, maximum aggregate size, and length and diameter of considered fibers. Li, Huang [19] reported enhanced mechanical characteristics and denser particle density of UHPC due to improved hydration by incorporating limestone powder. Huang and Cao [20] incorporated nano-CaCO_3_ as a binder component in UHPC and reported 17% enhanced compressive strength. Similar results are also reported in other studies [8, 21]. Soliman and Tagnit-Hamou [22] analyzed the incorporation of fine glass powder in UHPC to partially replace silica fume. The study concluded with attaining 235 MPa and 220 MPa compressive strength after steam curing for two days by partially substituting silica fume with 30% and 50% of fine glass powder content, respectively. Further, the incorporation of recycled glass powder as a partial replacement for quartz powder, quartz sand and cement has also been reported in the literature [23, 24]. All these studies showed the significance of using SCM as partial replacement of silica-fume and cement in UHPC to improve its flowability and compressive strength.

Though, the important characteristics of UHPC having SCMs require to be evaluated through experimentation due to the uncertain combination impacts of various materials and their contents being considered for the respective mix. The laboratory experimentation procedures usually consume excessive time, cost, and labor [2]. To reduce the experimental methods for evaluating the UHPC properties and mix design, machine learning models may be successfully applied to predict its properties [2, 25, 26]. With the incorporation of considerable SCMs volume in UHPC, the conventional regression approaches cannot attain the desired level of prediction accuracy [25]. In the recent past, nature encouraged the use of computational Artificial Intelligence (AI) approaches to resolve real-world issues because of its enormous capability to interpret co-relations between input and output parameters that are unknown, too difficult for the formulation, non-linear or representing its efficiency in resolving complex civil engineering problems [27].

Hence, applying AI-based machine learning (ML) approaches can help solve numerous issues with high complexity in different civil engineering fields [28, 29]. ML approaches can be applied to predict an ultimate result by considering a dataset of input variables [28]. Likewise, in this work, two ML techniques, i.e., a standalone approach (single model-based) and ensemble AdaBoost and Bagging algorithms, are employed to predict UHPC characteristics. As per the literature, the effectiveness in predicting performance from ensemble modelling approaches is more than an individual algorithm. The employment of ML approaches is done by Chaabene, Flah [30] for the prediction of concrete mechanical properties. Similarly, the properties of different types of concrete, such as High-Performance Concrete (HPC) [31–35], self-healing concrete [36], Recycled Aggregate Concrete (RAC) [37–40], phase change materials-integrated concrete[41] etc. have also been predicted by the employment of ML approaches in the literature. The employment of ML techniques is done by Han, Gui [32] for the prediction of HPC flowability. The input variables comprised of cement, water, coarse aggregates, age, sand, GGBFS, fly ash, and five combinations of variables were taken. The developed algorithms predicted the highly accurate flowability for HPC. In this way, the application of ML techniques for determining the concrete properties would be the basis to conserve time and cost of future researchers.

## 2. Research significance

The application of ML approaches results in the possible estimation of properties for different concrete types with the least variation in trials, which otherwise consume considerable time, effort, and cost for casting, curing and testing in experimental investigation. Hence, it is necessary to establish algorithms based on data modelling along with identifying closely related independent variables and a quick decrease in input matrix dimensions. The employment of Artificial Intelligence (AI) techniques is therefore gaining much attention in civil engineering to predict concrete behavior. It is an alternative approach for predicting the compressive strength and flowability of UHPC to save the effort, time, and cost involved in experimental procedures. In this work, the employment of an individual machine learning algorithm and different ensembled machine learning approaches for predicting UHPC compressive strength and flowability is done. Decision Tree (DT) is considered an individual ML algorithm, whereas Gradient Boosting (GB) and Bootstrap Aggregating (BA) are taken as ensembled ML algorithms. Moreover, statistical checks are applied to evaluate the considered models, and a comparison of the performance of these models is also made. Subsequently, for the prediction of UHPC properties, a recommendation for a model with higher precision is also made based on its performance evaluated by the above-mentioned statistical checks. In addition to that, in this study, the feature importance plotting by applying a game theory approach named SHapley Additive exPlanations (SHAP) is also done for having an in-depth exploration of UHPC mix design to attain strength through non-linear behavior as well as for detailed description regarding the contribution of input variables. Overall, UHPC structure properties are correlated in this study by employing interpretable ML approaches via feature importance. Accordingly, the current research would benefit the development of concrete structures.

## 3. Data set

**Fig 1:**
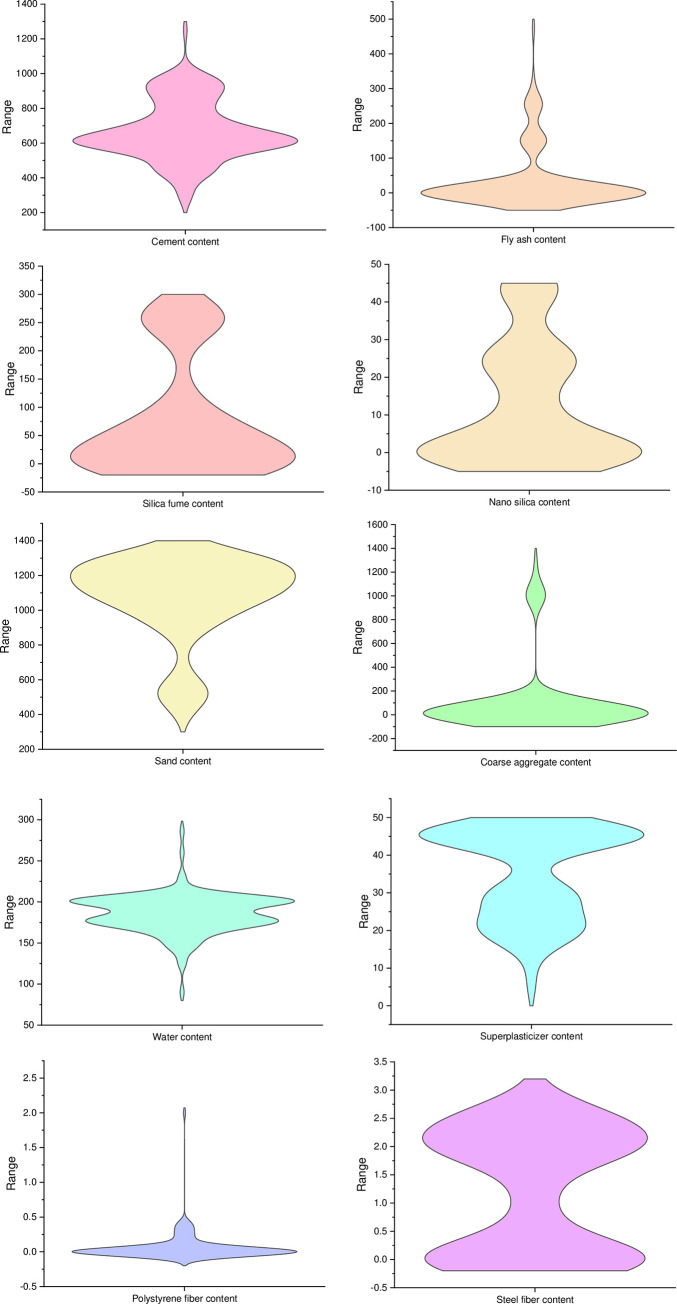
Violin plot for flowability input parameters.

The database for predicting UHPC flowability is extracted from the literature [42] comprising 135 mix combinations with 21 input parameters (Fig 1). The dataset consists of the contents of cement (Kg/m^3^), fly-ash (Kg/m^3^), silica-fume (Kg/m^3^), slag (Kg/m^3^), nano-silica (Kg/m^3^), quartz powder (Kg/m^3^), limestone powder (Kg/m^3^), sand (Kg/m^3^), coarse aggregates (Kg/m^3^), super-plasticizers (Kg/m^3^), steel fiber (%), polystyrene fibers (%) and water(Kg/m^3^), type, strength-class and compressive strength (MPa) of cement, maximum aggregate size (mm), and length (mm) and diameter (μm & mm) of polystyrene fiber and steel fiber. The output parameters, i.e., flowability (cm) and prediction variables, are based on these input factors.

**Fig 2:**
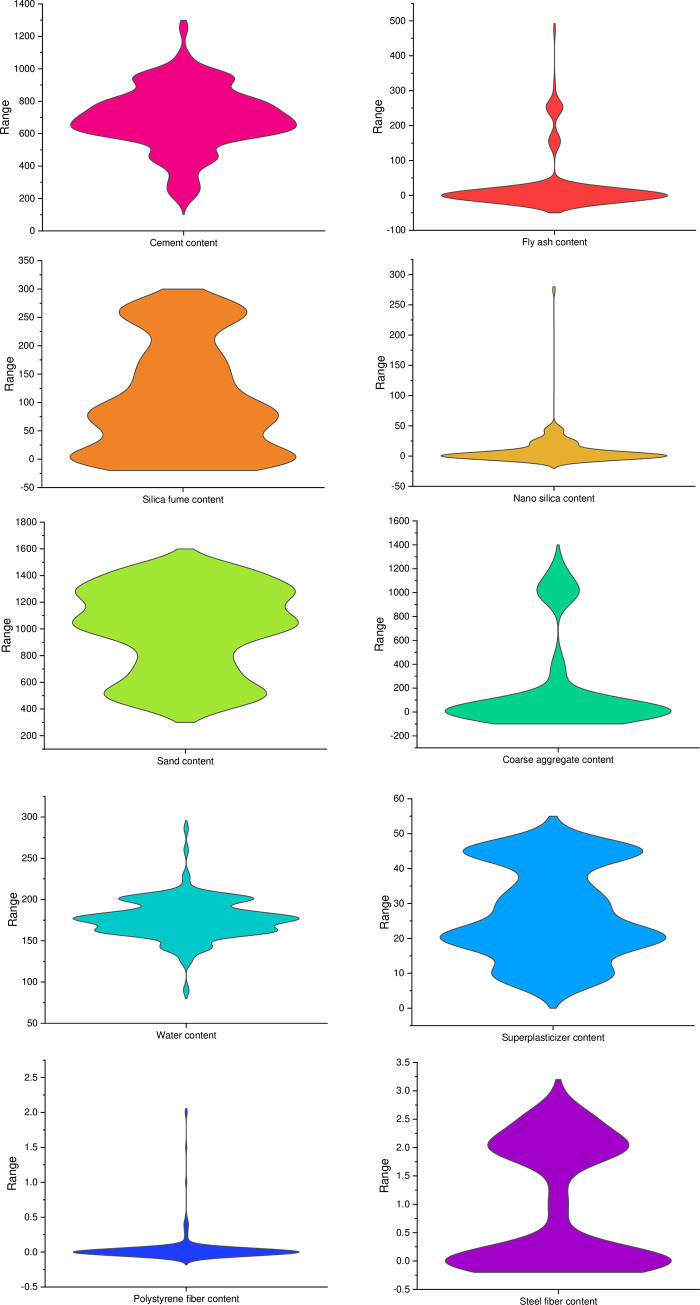
Violin plot for compressive strength input parameters.

**Fig 3:**
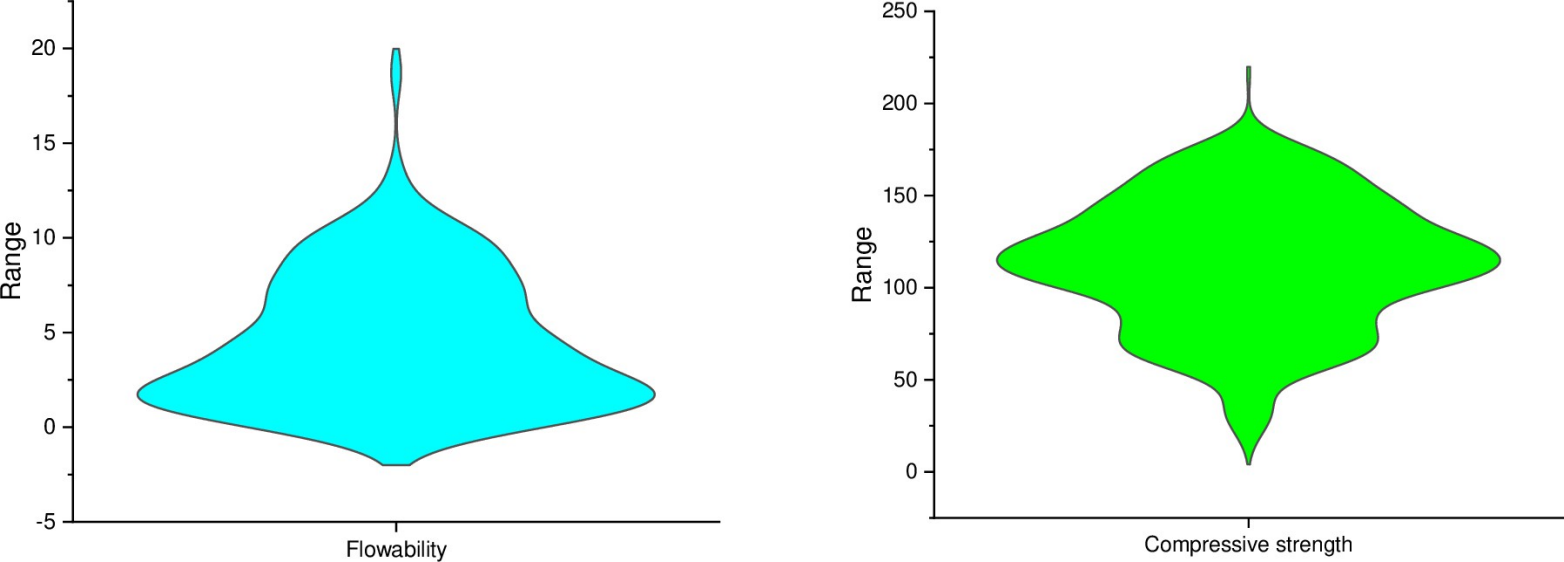
Violin plot for flowability and compressive strength (i.e., output parameters).

The input dataset for the prediction of UHPC compressive strength is extracted from the literature [42], which comprises 626 mix combinations with 21 input variables (Fig 2). The considered input variables are; cement content (Kg/m^3^), fly-ash content (Kg/m^3^), silica-fume content (Kg/m^3^), slag content (Kg/m^3^), nano-silica content (Kg/m^3^), quartz powder content (Kg/m^3^), limestone powder content (Kg/m^3^), sand content (Kg/m^3^), coarse aggregates content (Kg/m^3^), super-plasticizers content (Kg/m^3^), steel fiber content (%), polystyrene fibers content (%), water content (Kg/m^3^), cement type, cement strength class, cement compressive strength (MPa), maximum aggregate size (mm), steel fiber length (mm), steel fiber diameter (mm), polystyrene fiber length (mm) and polystyrene fiber diameter (μm). The output parameters, i.e., compressive strength (MPa) and prediction variables, are based on these input factors. Fig 3 depicts the violin plot for both the output parameters, i.e., UHPC flowability and compressive strength. Anaconda software "Spyder" with Python scripting is applied to predict UHPC compressive strength and flowability.

## 4. Results and analysis

### 4.1. Decision Tree (DT)

**Fig 4:**
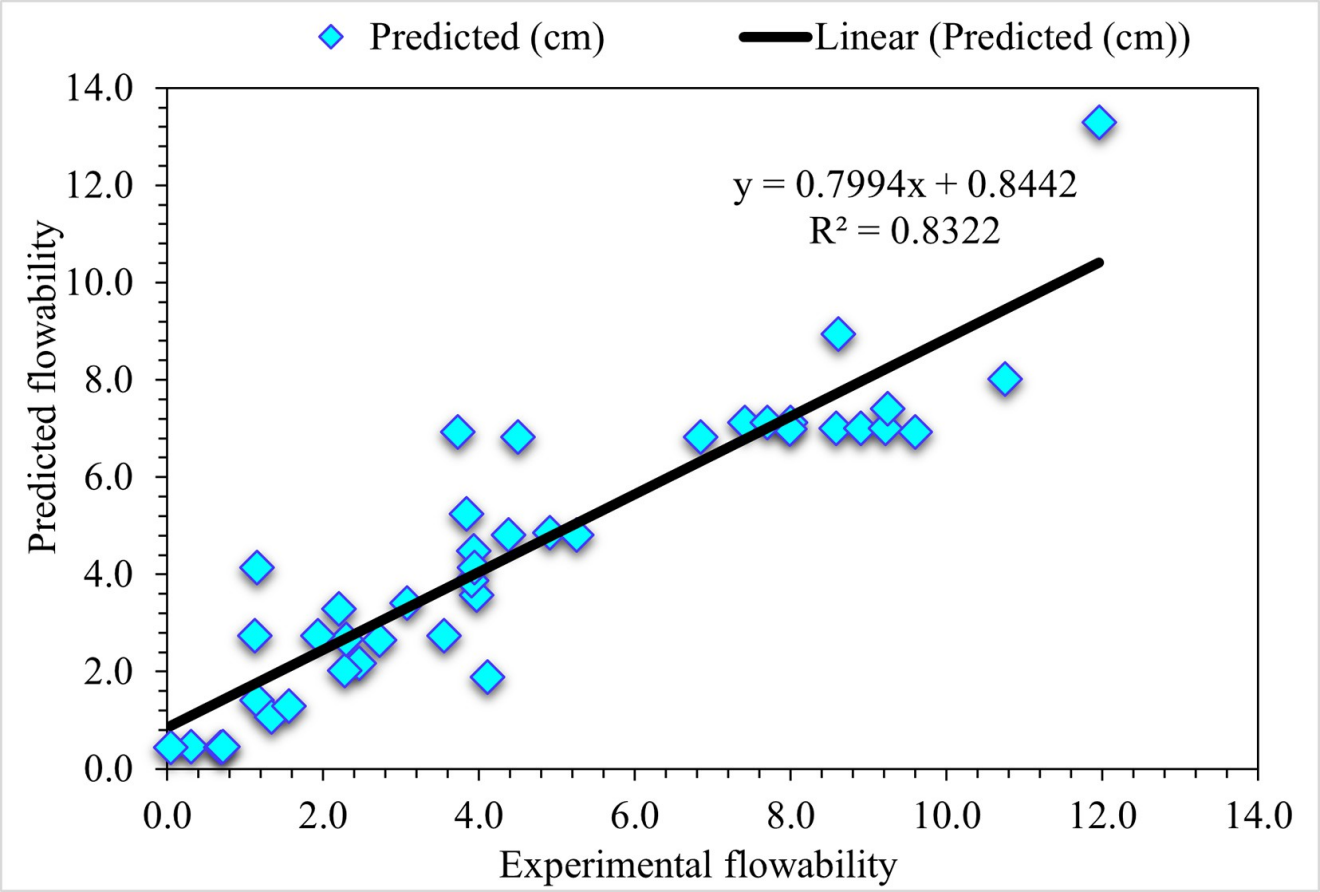
DT predicted and experimental values—UHPC flowability.

**Fig 5:**
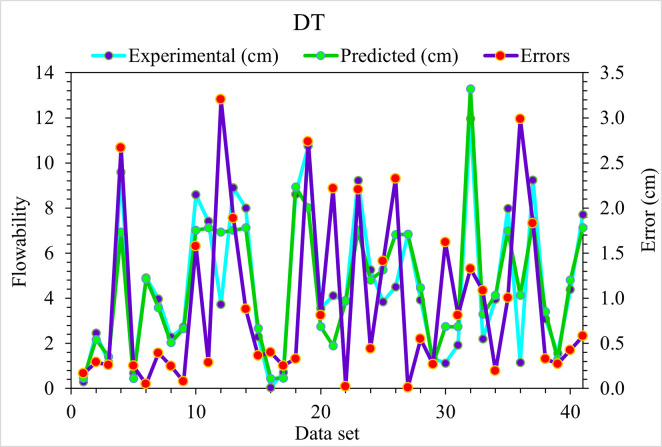
DT predicted and experimental error values—UHPC flowability.

Fig 4 compares experimental and predicted values for UHPC flowability obtained from the DT model. It is observed that the DT model depicts a reliably predicted outcome having the least variation in the case of UHPC flowability. The adequacy of the DT algorithm is depicted from an acceptable R^2^ value, i.e., 0.83. Fig 5 shows the DT error distribution against experimental and predicted values for the flowability of UHPC. It may be noted that, for UHPC flowability, 3.20 cm is the average error value. Here, 17.1% of values are above 2 cm, 17.1% lie from 1 to 2 cm, and 65.9% are less than 1 cm.

**Fig 6:**
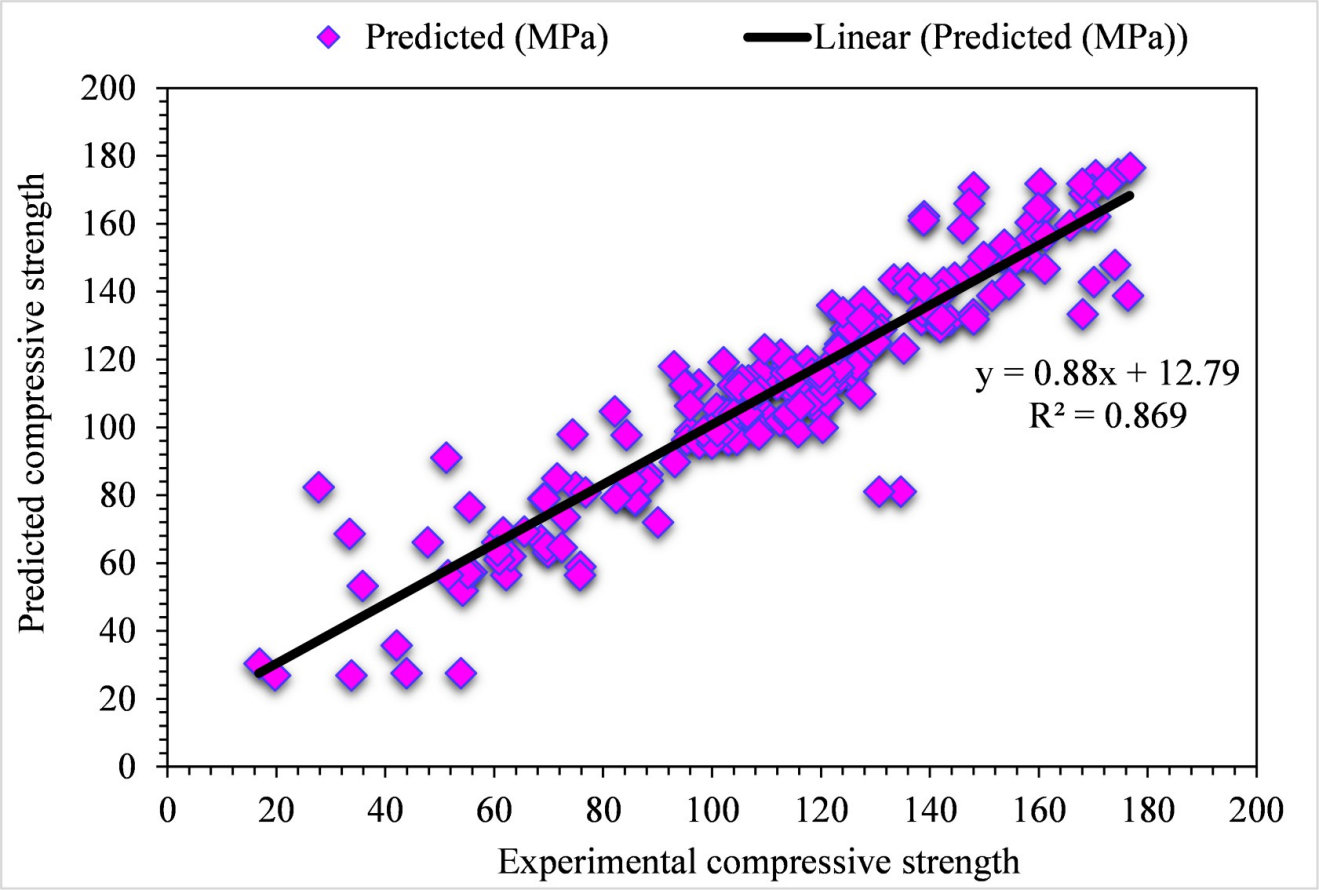
DT predicted and experimental values—UHPC compressive strength.

**Fig 7:**
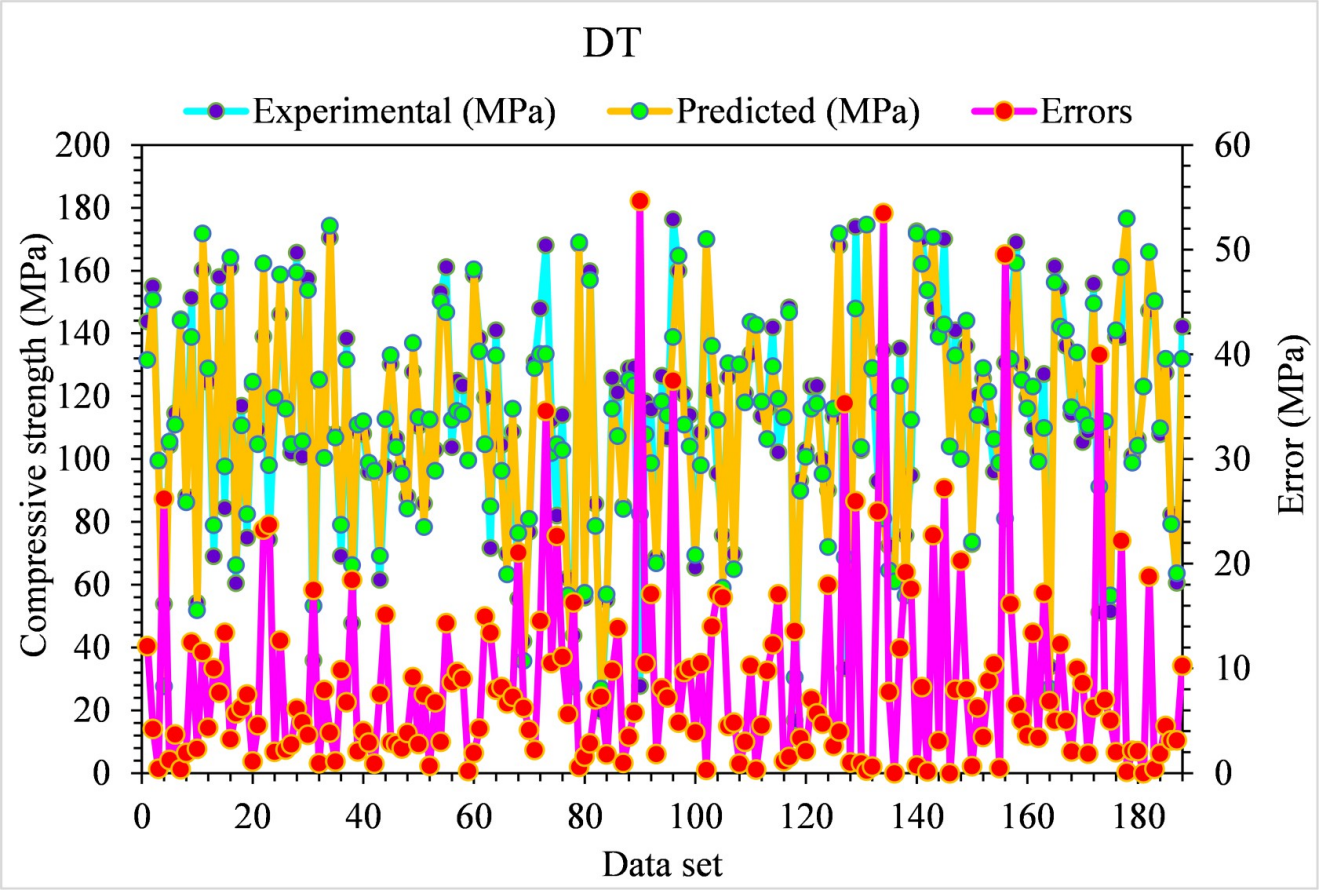
DT predicted and experimental error values–UHPC compressive strength.

However, for the compressive strength of UHPC, the experimental and DT predicted values are shown in Fig 6. Here, in this case, the least accuracy can be demonstrated from the 0.81 R^2^ value. But the DT-predicted results for compressive strength of UHPC fall in an acceptable range. The experimental and DT-predicted error distribution values for the compressive strength of UHPC are shown in Fig 7. Here, 29% of error values fall below 1 MPa, 66% lie from 1 to 5 MPa, and the remaining 5% are above 5 MPa.

### 4.2. Gradient Boosting (GB)

**Fig 8:**
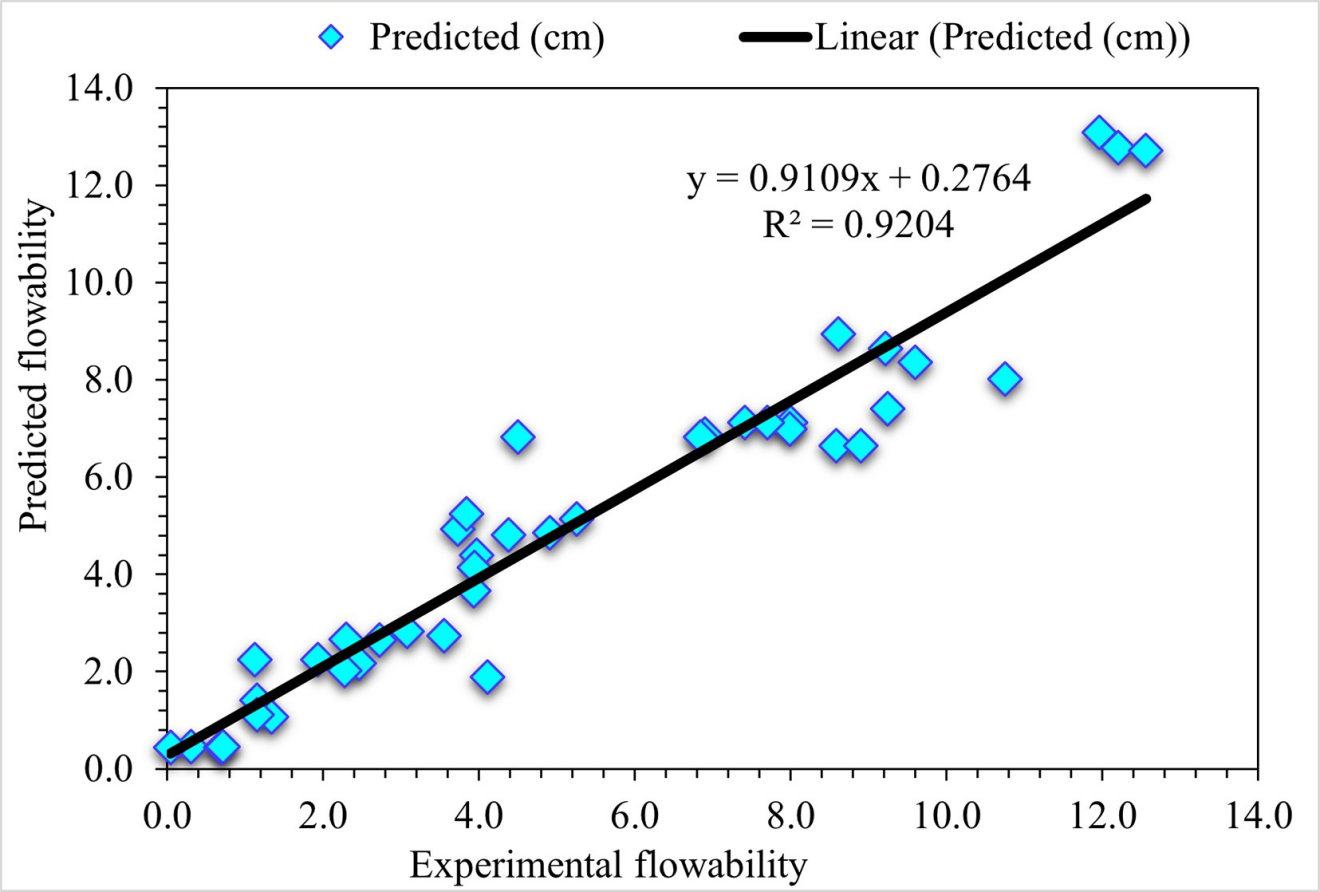
GB predicted and experimental values—UHPC flowability.

**Fig 9:**
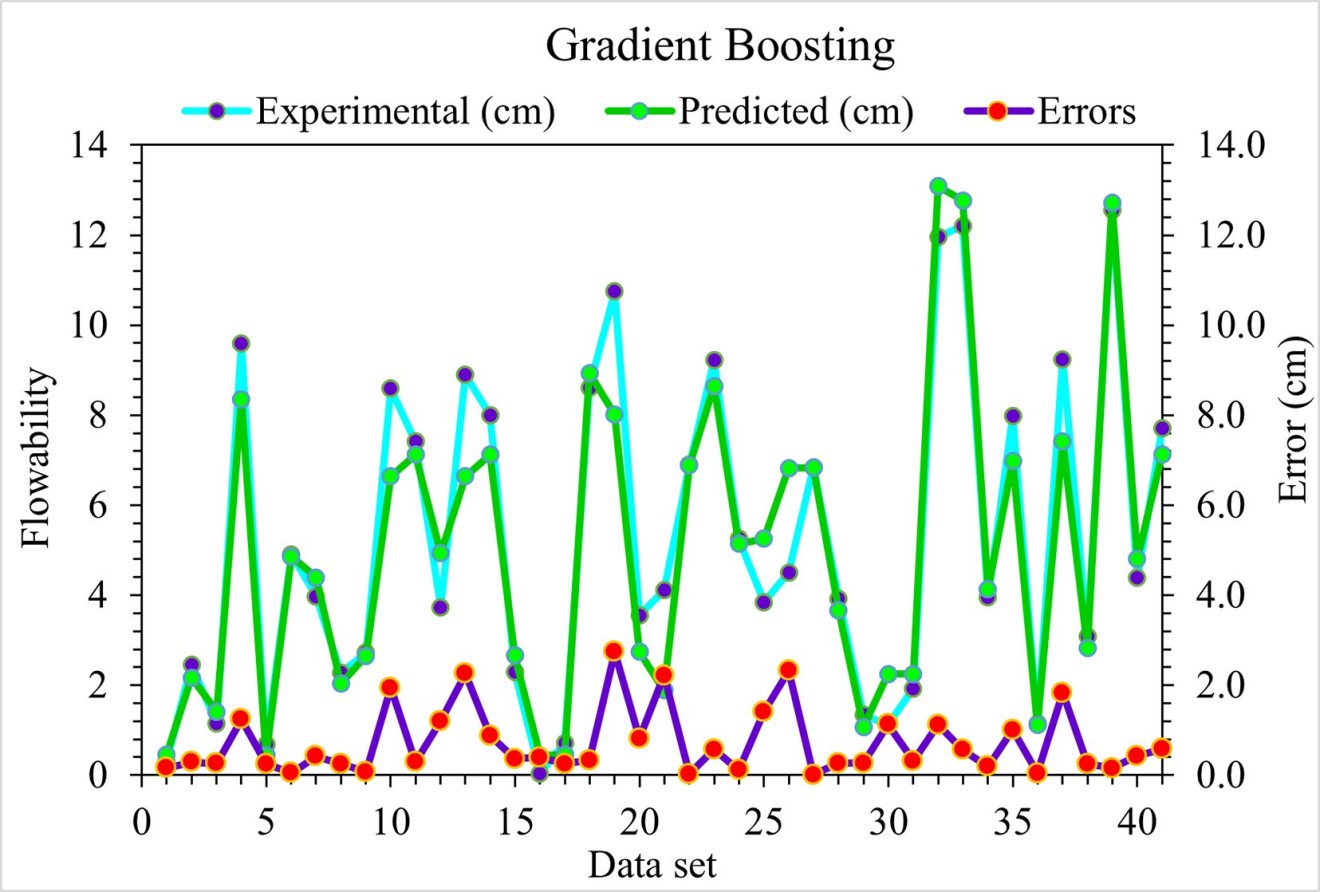
GB predicted and experimental error values—UHPC flowability.

The predicted (GB) model and experimental outcomes for flowability of UHPC are presented in Fig 8. The R^2^ value of 0.92 in the case of GB depicts more precise results than the DT algorithm. The error distribution in the case of experimental and GB-predicted values for UHPC flowability is shown in Fig 9. It may be noted here that 9.8% of error values are higher than 2 cm, 17.1% are from 1 to 2 cm, and 73.2% are less than 1 cm. The lesser error and higher R^2^ values depict a higher accuracy level of the GB algorithm than DT.

**Fig 10:**
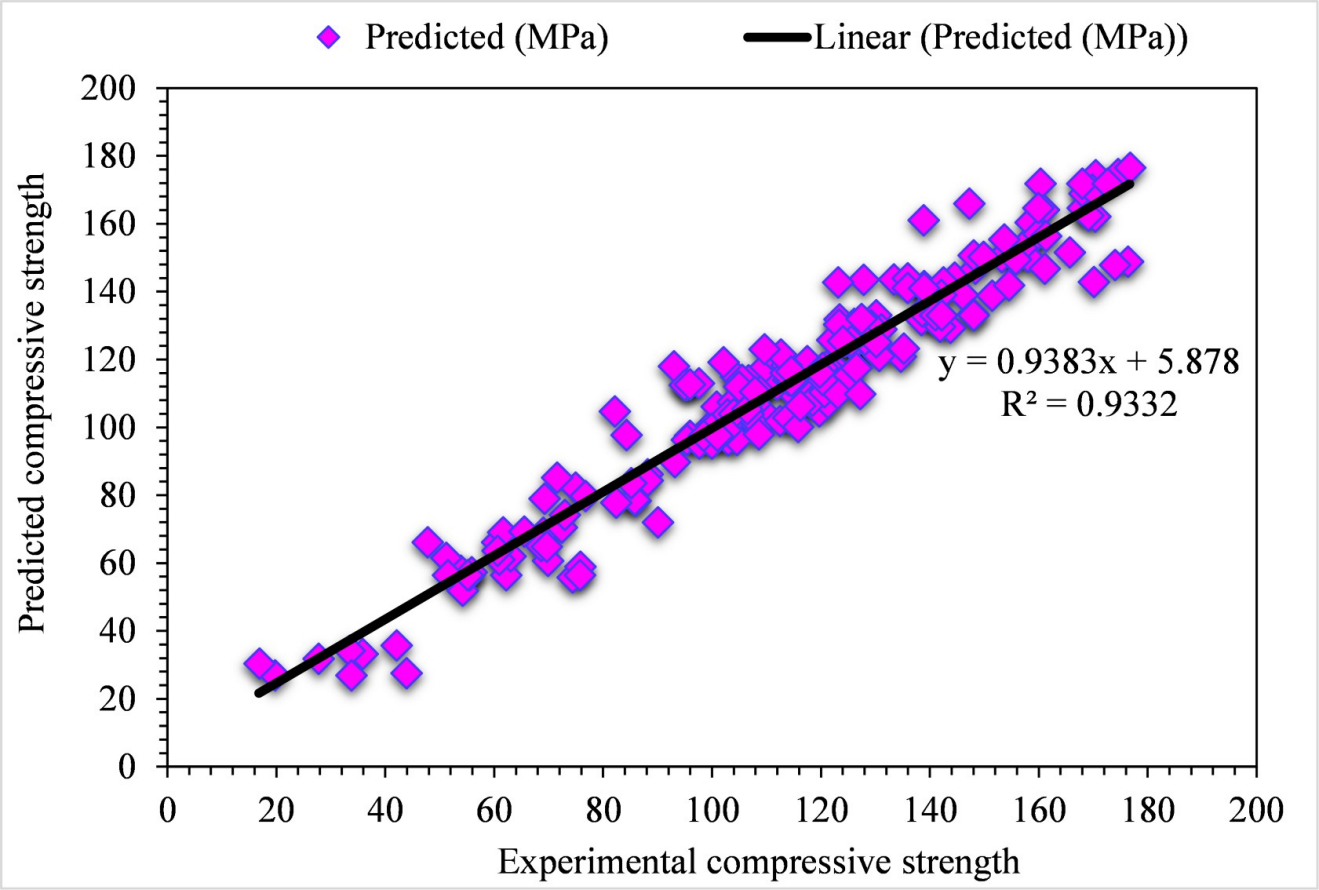
GB predicted and experimental values—UHPC compressive strength.

**Fig 11:**
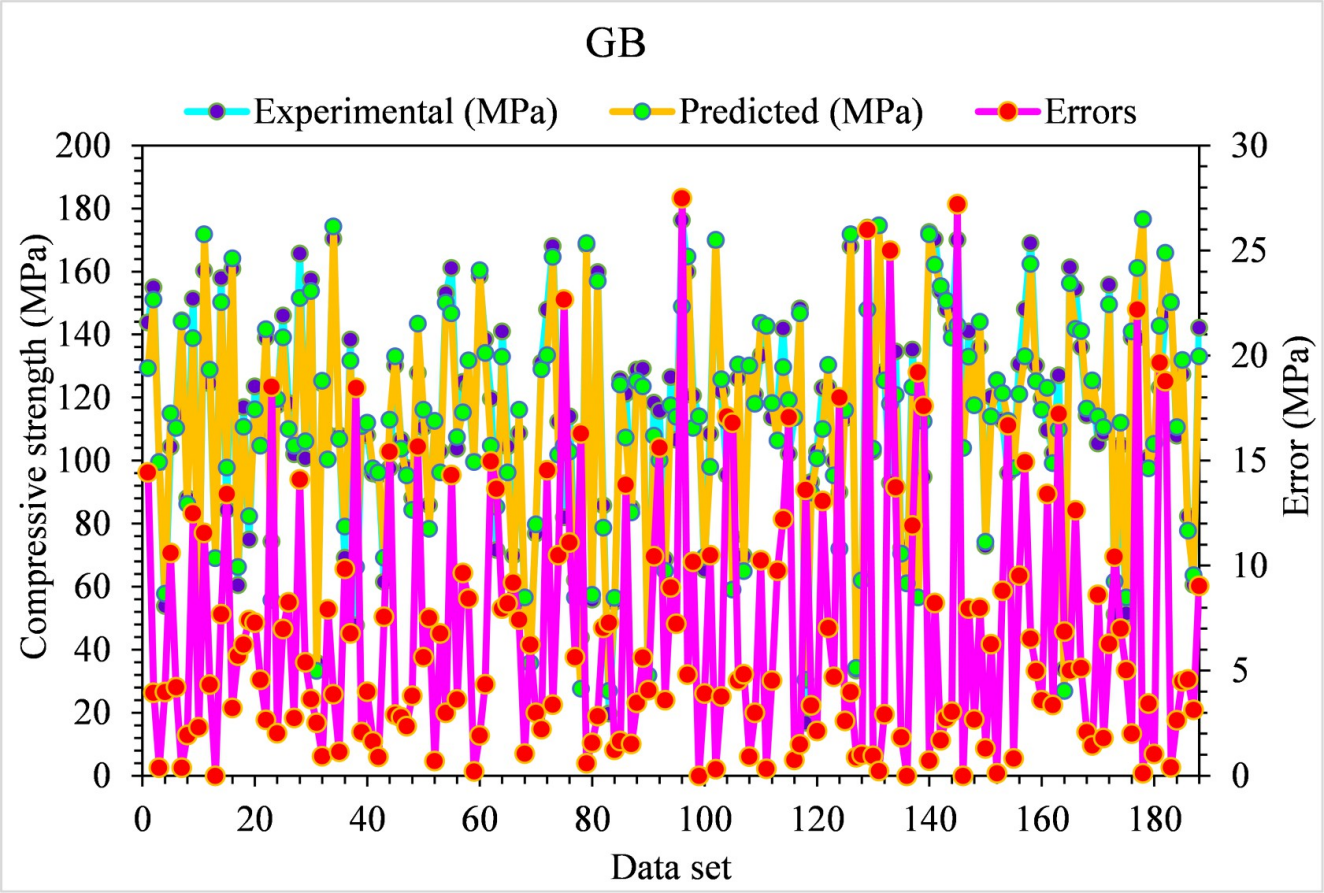
GB predicted and experimental error values–UHPC compressive strength.

The experimental and GB model predicted values for the compressive strength of UHPC are shown in Fig 10. The GB depicts lesser variation in error for compressive strength of UHPC and preferred predicted results. The appropriateness of the GB algorithm is shown by an acceptable R^2^ value, i.e., 0.94. The error distribution of experimental and GB predicted values for the compressive strength of UHPC is presented in Fig 11. In the case of UHPC compressive strength, the average value for error is 1.57 MPa. Here, not a single value is above 5 MPa, 53% of values are from 1 to 5 MPa, and 47% are less than 1 MPa.

### 4.3. Bootstrap aggregating (BA)

**Fig 12:**
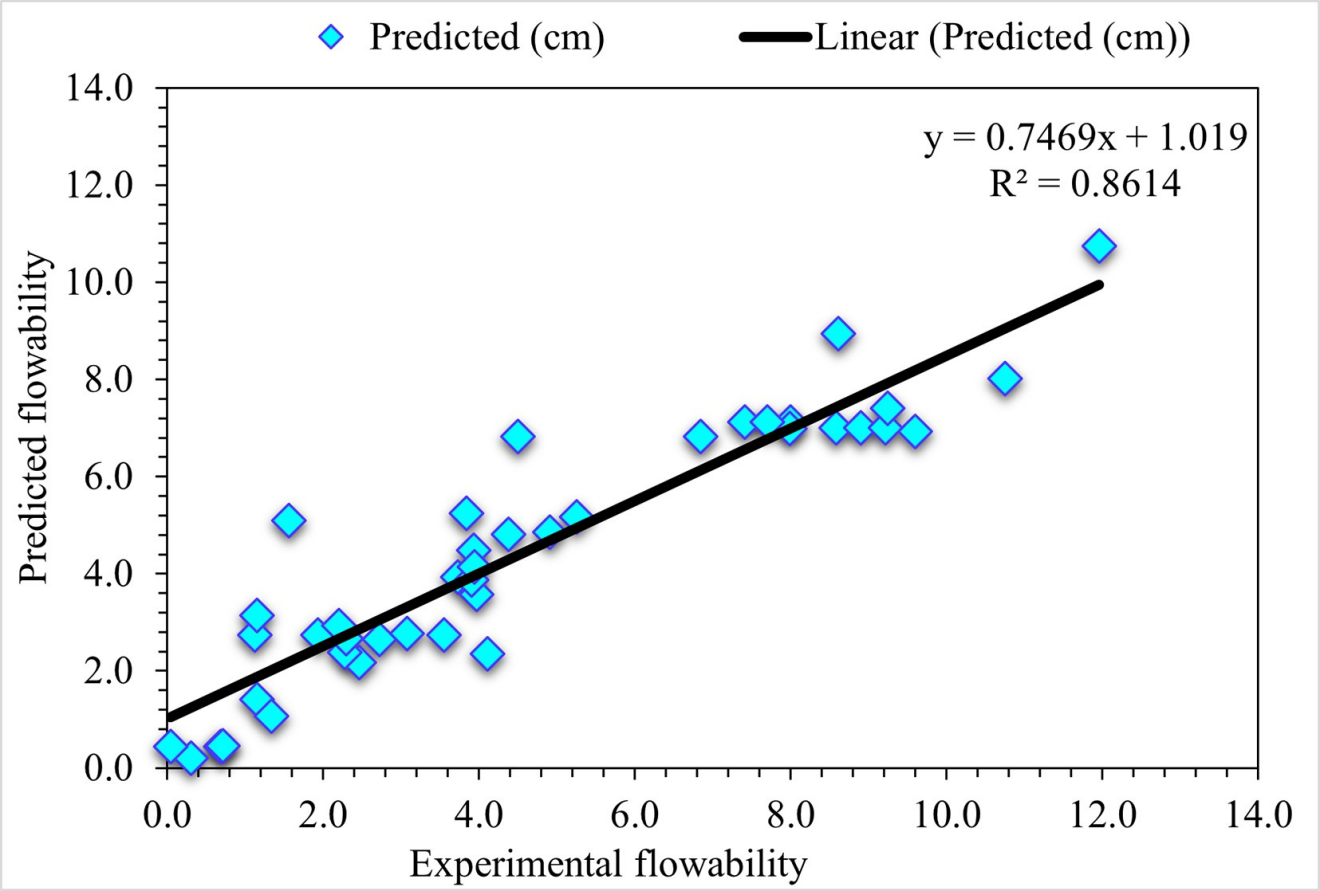
BA predicted and experimental values—UHPC flowability.

**Fig 13:**
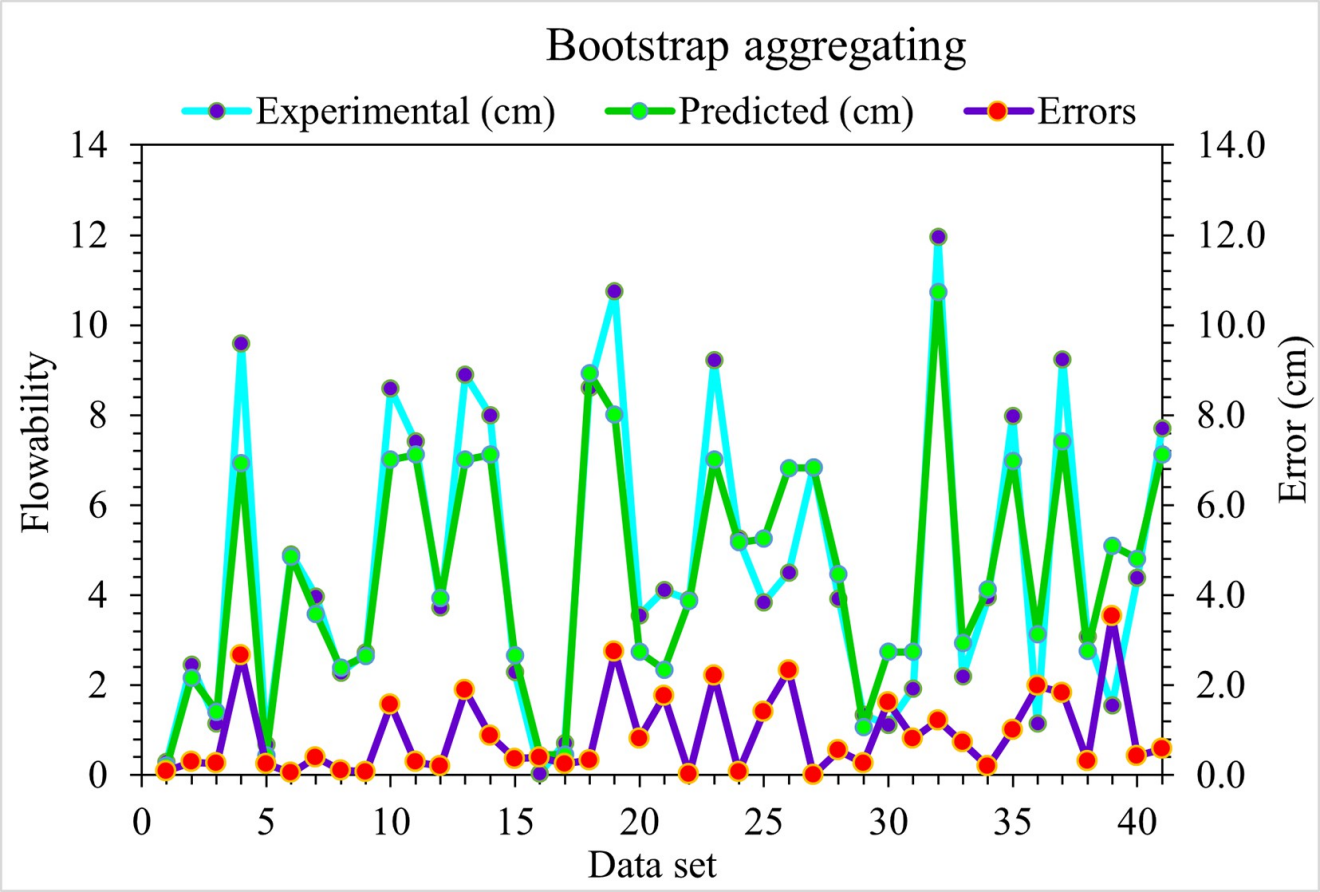
BA predicted and experimental error values—UHPC flowability.

The experimental and BA predicted results in the case of UHPC flowability are shown in Fig 12. Comparatively lesser precise results are depicted from an R^2^ value of 0.86 than the employed ensembled GB algorithm. However, the predicted outcomes for flowability of UHPC in the BA algorithm are comparatively superior to individual DT algorithms. Fig 13 represents the experimental and BA predicted error distribution values for the flowability of UHPC. Here, 12.2% of values are more than 2 cm, 19.5% lie between 1 to 2 cm, and the remaining 68.3% are less than 1 cm. The BA R^2^ and error values for the flowability of UHPC are less accurate than the DT model. However, the R^2^ and error values in the case of ensembled ML models are more adequate than DT. Hence, this result shows that GB’s prediction results are more accurate than other considered algorithms.

**Fig 14:**
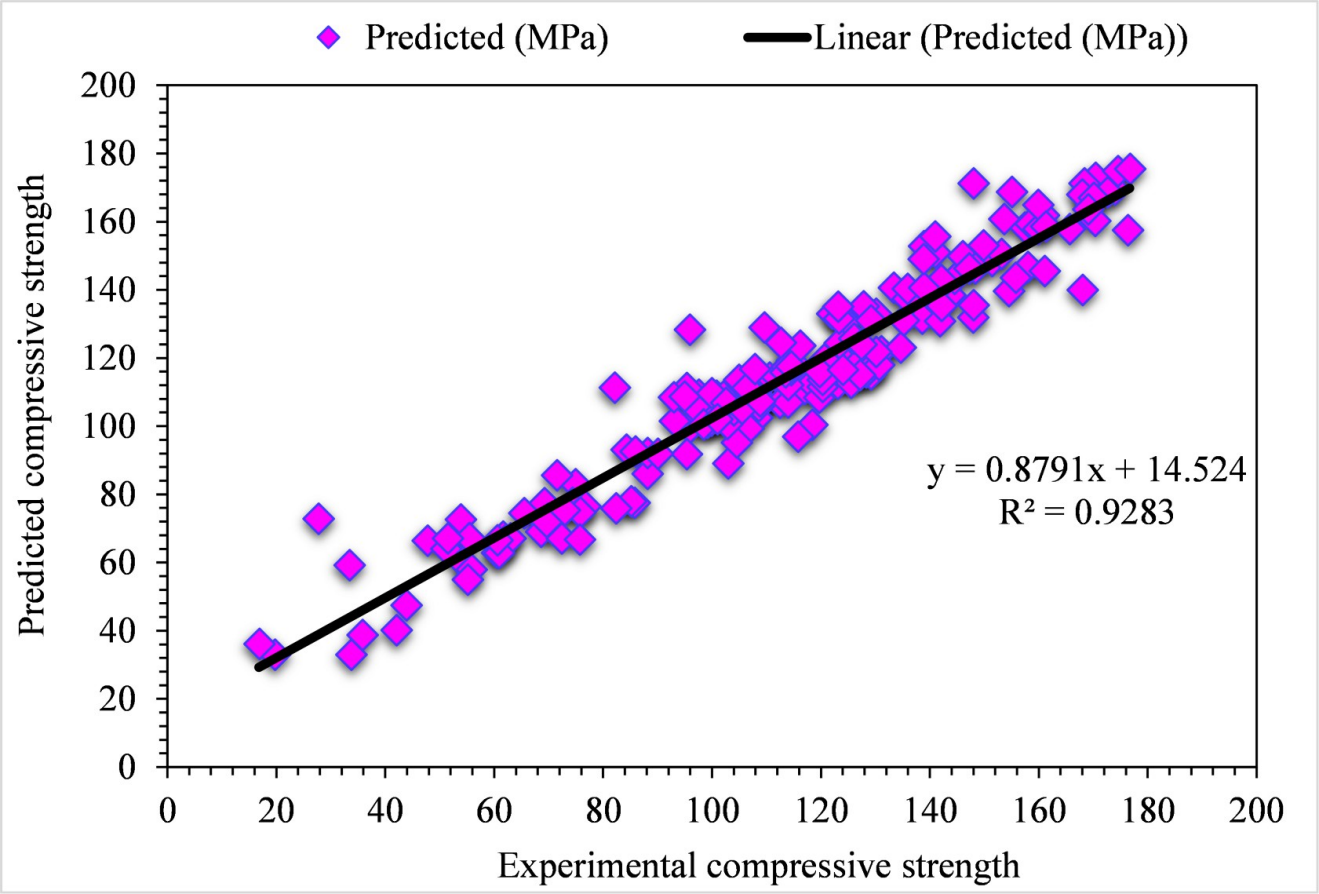
BA predicted and experimental values—UHPC compressive strength.

**Fig 15:**
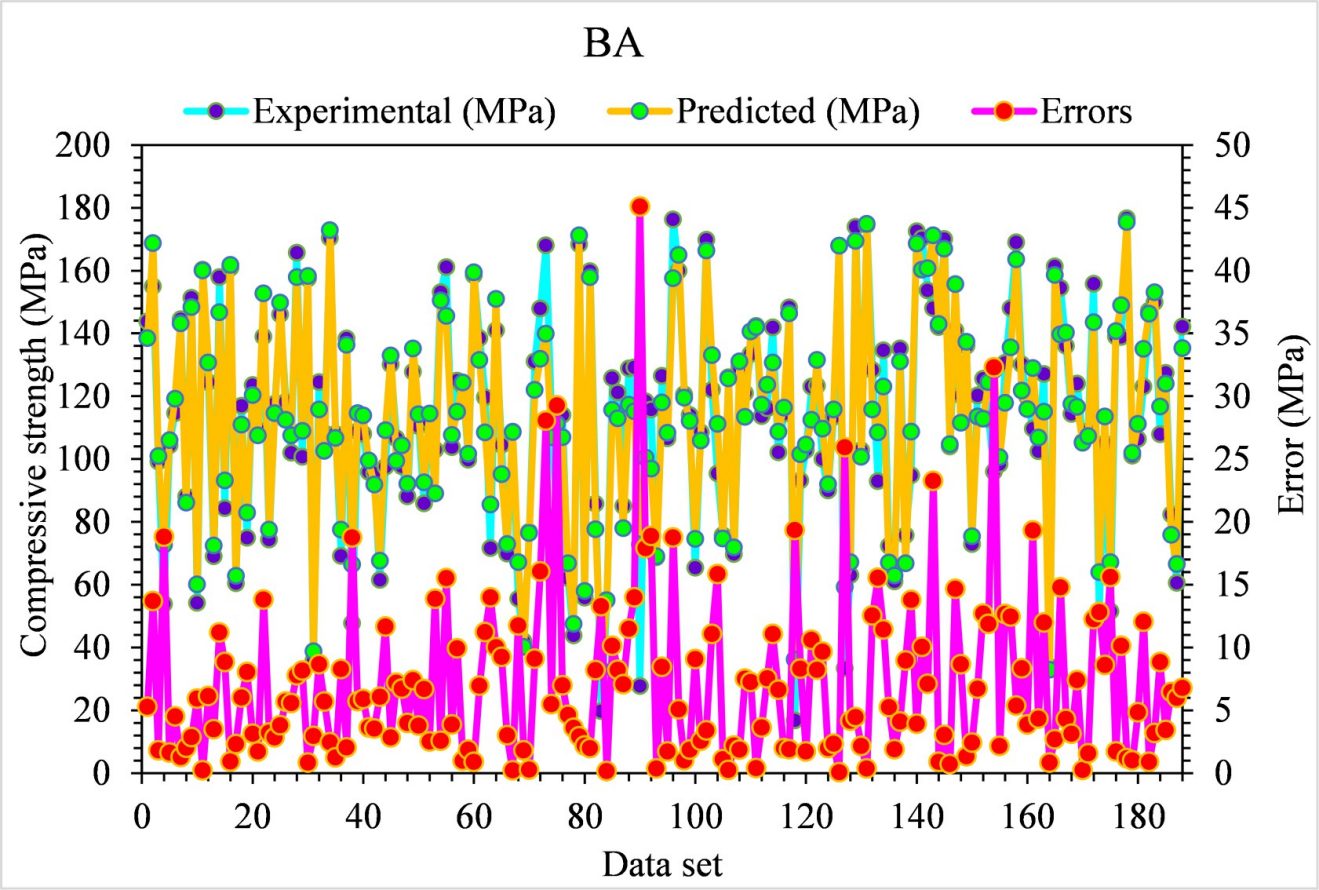
BA predicted and experimental error values–UHPC compressive strength.

The experimental and BA-predicted outcome values for the compressive strength of UHPC are shown in Fig 14. The 0.93 R^2^ value for the BA algorithm depicts results with lesser precision than the GB model. The experimental BA predicted error distribution values for the compressive strength of UHPC are presented in Fig 15. It may be noted that only 4% of values are above 5 MPa, 62% of values fall from 1 to 5 MPa, and the remaining 33% of values are below 1 MPa. The lesser error and higher R^2^ values show the high precision of the GB algorithm compared to DT. In contrast, the attained error and R^2^ values in the BA ensembled ML algorithm is appropriate. So, this result shows that GB prediction outcomes have more accuracy than all other considered models.

### 4.4. Comparison of all models

**Fig 16:**
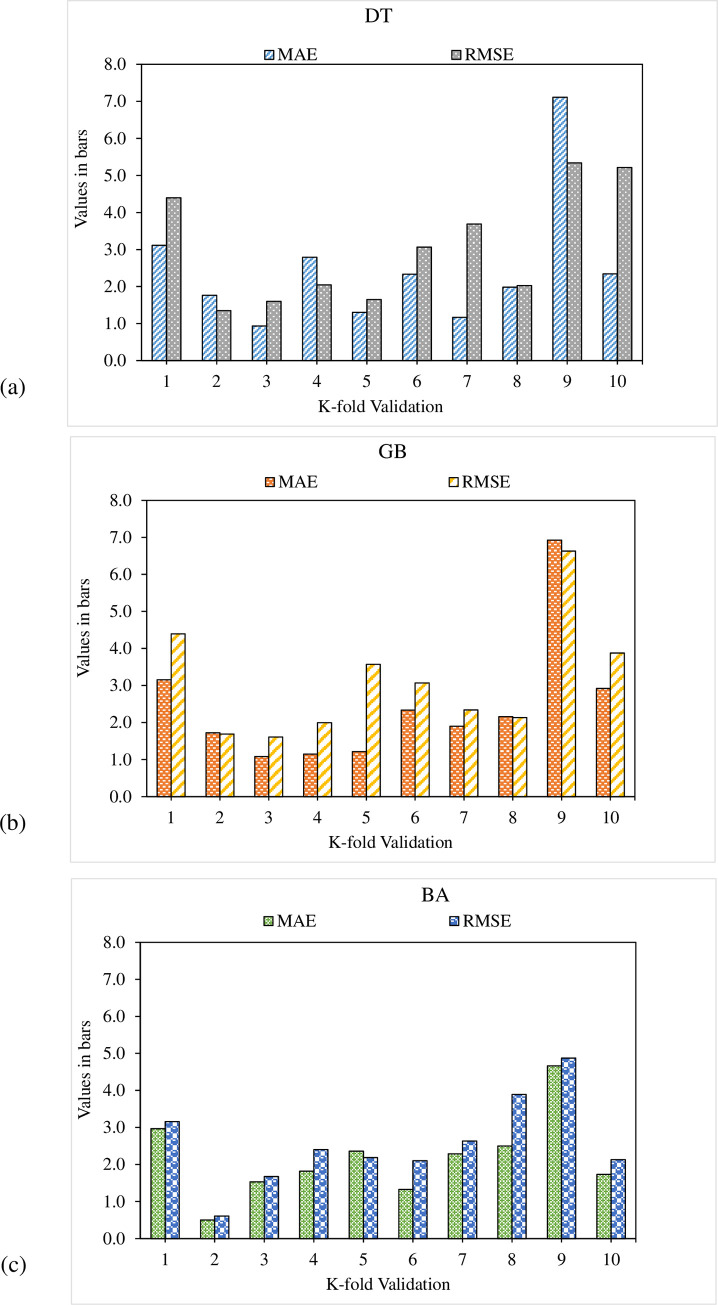
Flowability statistical representation: (a) DT; (b) GB; (c) BA.

**Fig 17:**
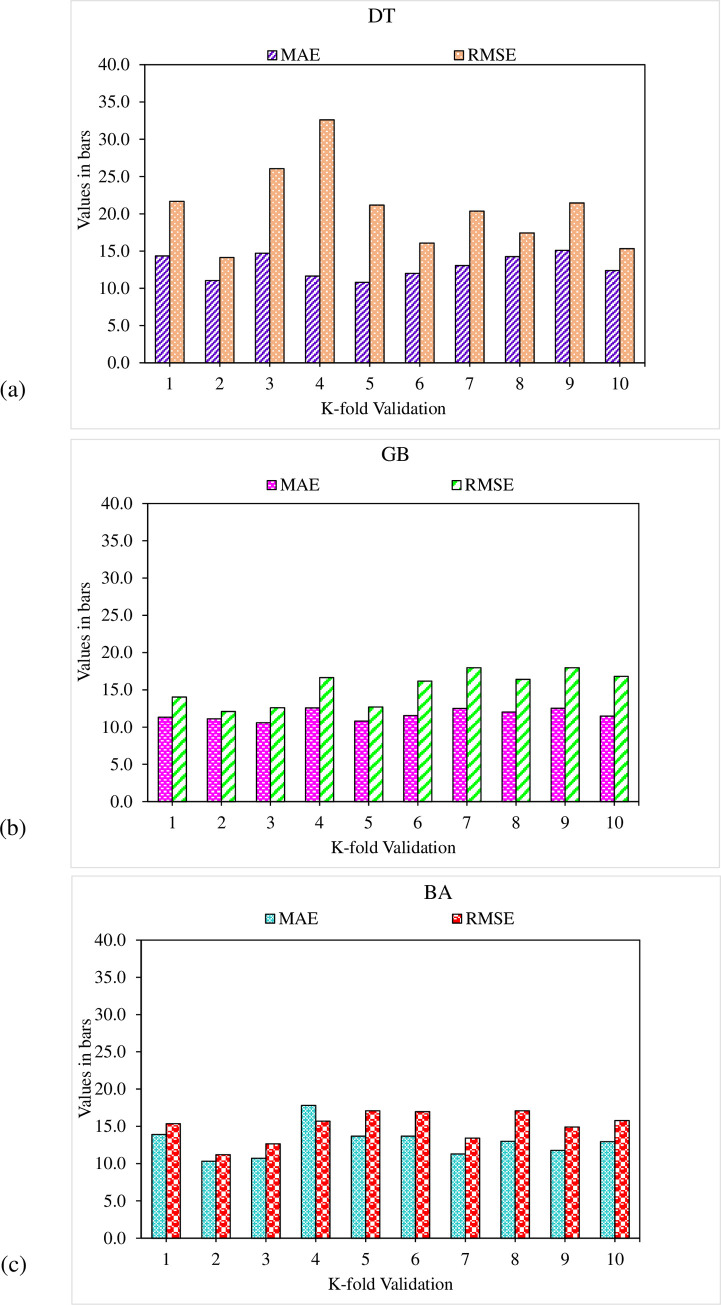
Compressive strength statistical representation: (a) DT; (b) GB; (c) BA.

The employment of the k-fold cross-validation method evaluates the validity of all the considered models. In literature, the model’s performance is reported to be assessed with the help of statistical checks [43–46]. In the k-fold cross-validation approach, the entire data is split into ten sub-groups to disperse it randomly. The same process is repeated ten times to attain acceptable results. Table 1 illustrates the applied statistical checks against all the considered models. The MAE values for UHPC flowability in the DT, GB, and BA algorithms are 1.0 cm, 0.9 cm and 0.7 cm, respectively, as illustrated in Fig 16(A)–16(C). However, for UHPC compressive strength, the RMSE values in the DT, GB and BA algorithms are 12.9 MPa, 9.7 MPa and 9.2 MPa, respectively, as illustrated in Fig 17(A)–17(C). It may be noted that RMSE and MAE value in the case of GB is comparatively lesser than other considered algorithms with lesser values of error for both the compressive strength and flowability of UHPC.

**Table 1:** Statistical evaluation for DT, GB and BA algorithms.

Statistical Checks	Parameters					
	Compressive Strength			Flowability		
	DT Algorithm	GB Algorithm	BA Algorithm	DT Algorithm	GB Algorithm	BA Algorithm
R^2^	0.86	0.93	0.92	0.83	0.92	0.86
RMSE	12.9 MPa	9.2 MPa	9.7 MPa	1.7 cm	1.2 cm	1.6 cm
MAE	8.8 MPa	7.0 MPa	7.2 MPa	1.0 cm	0.7 cm	0.9 cm

In the current study, the flowability of UHPC is predicted by the employment of ensembled ML approaches to have efficient and reliable outcomes. Consequently, the R^2^ value of 0.92 in the GB algorithm depicts the higher accuracy level in the flowability prediction for UHPC. The GB, an ensembled ML algorithm, shows better prediction performance for UHPC flowability by employing an optimized algorithm out of 20 sub-models, as illustrated in Fig 16. The superior accuracy of ensembled GB models is evident compared to other considered models.

**Fig 18:**
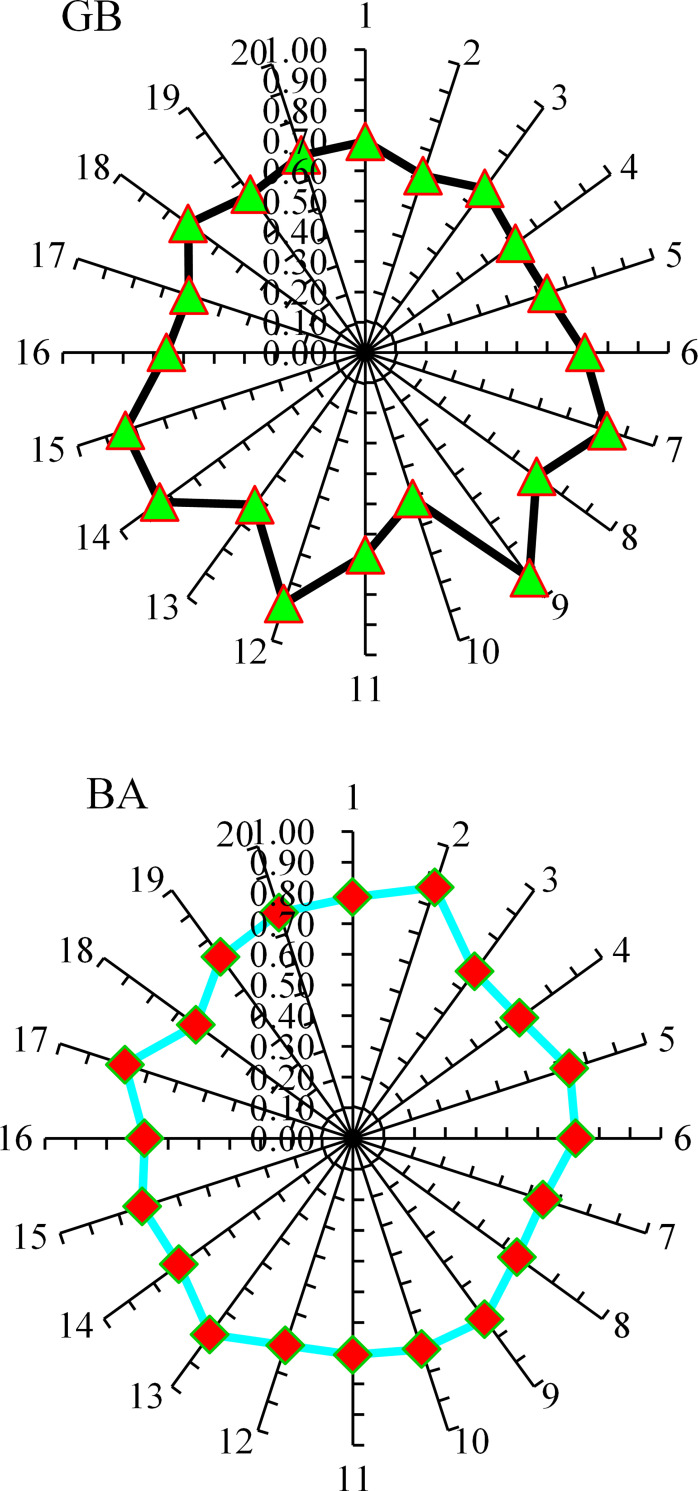
Sub-model results for flowability: (a) GB; (b) BA.

**Fig 19:**
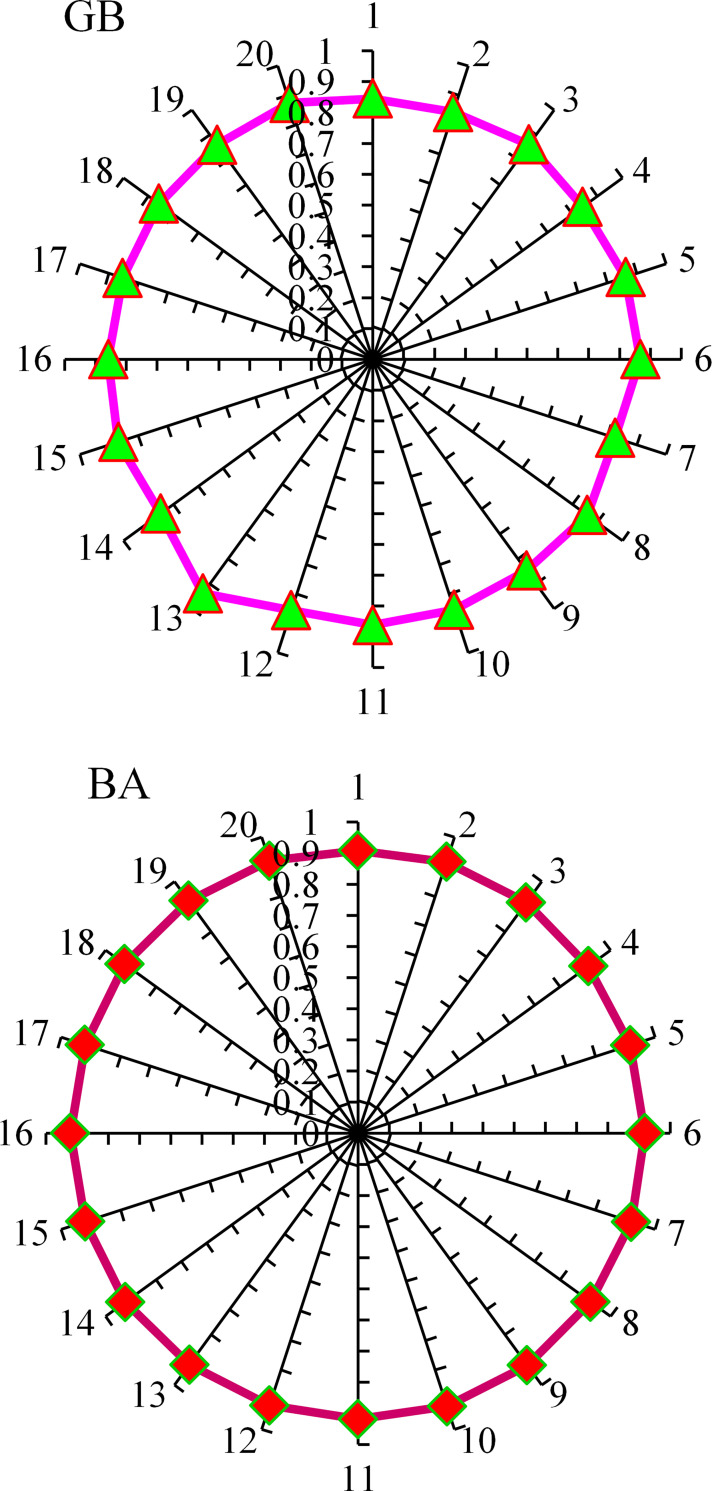
Sub-model results for compressive strength: (a) GB; (b) BA.

Further, in this work, the prediction for the compressive strength of UHPC is made by employing ensembled ML approaches to get efficient and reliable results. Here, in this case, the precision of the GB model for UHPC compressive strength is depicted from R^2^ value of 0.93. Here again, the performance of the optimized model out of 20 sub-models is better for the compressive strength prediction of UHPC via the ensembled GB ML model (Fig 17). Compared with other considered algorithms, in the case of UHPC compressive strength, the ensembled GB algorithms depict more precision and lesser error. The sub-models result in gradient boosting and bootstrap aggregation for UHPC flowability and compressive strength are shown in Figs 18 and 19, respectively.

## 5. Feature importance of ML models for flowability and compressive strength

**Fig 20:**
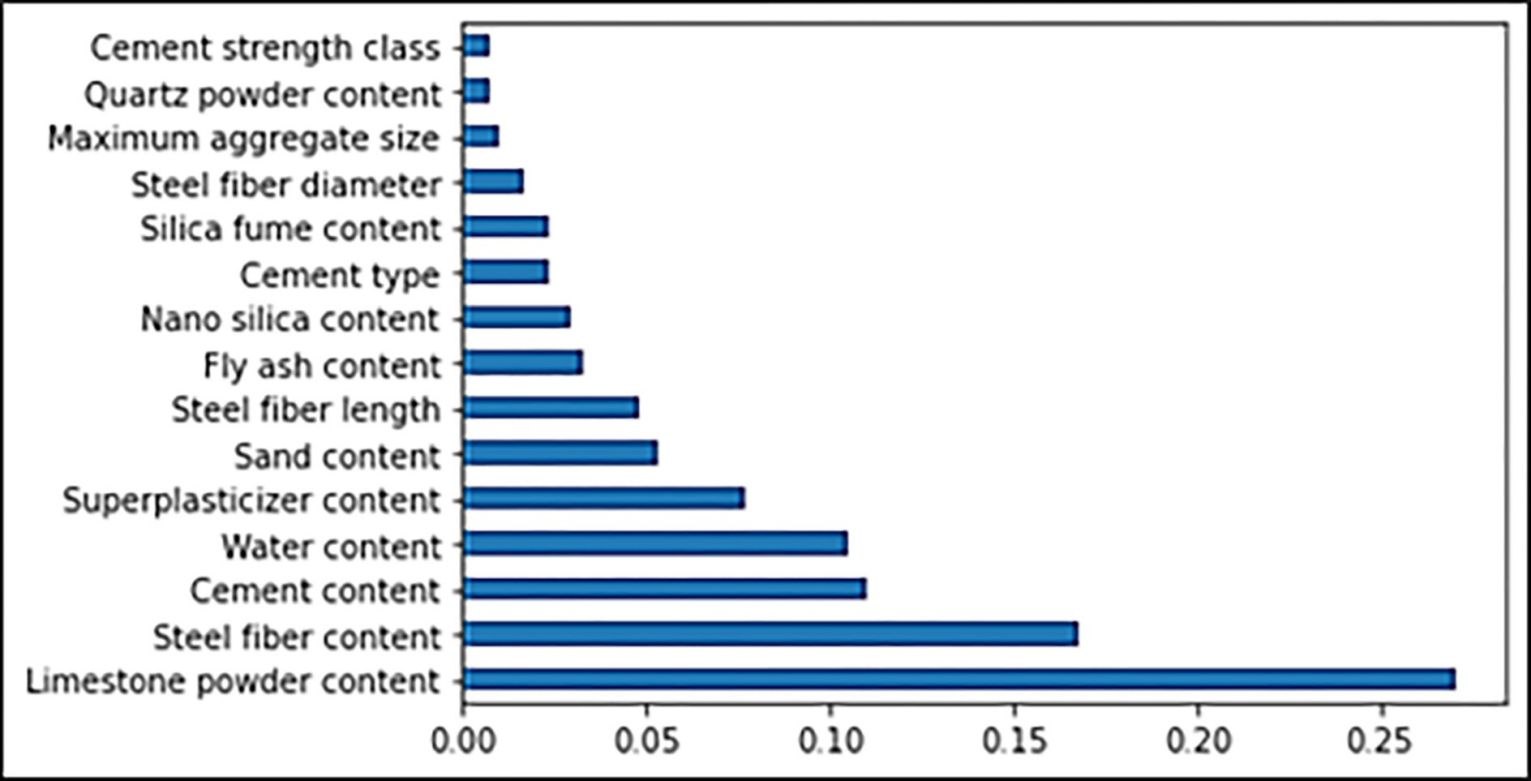
Feature importance of flowability.

**Fig 21:**
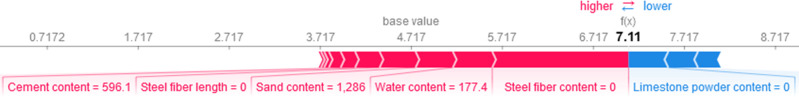
SHAP plot of flowability.

In the current work, a thorough explanation is also made for the employed ML models and the dependencies/interactions for all the input features. The features’ correlation importance for the flowability of UHPC is presented in Fig 20. It may be noted that the limestone powder feature has almost 0.27 value, which is the highest in UHPC flowability prediction. Further, this limestone powder feature shows a positive influence which means; by enhancing the content of limestone powder, the flowability of UHPC enhances. A similar trend in the case of limestone powder feature is also evident from the SHAP plot (Fig 21) for UHPC flowability. The steel fiber content feature is in second place in terms of the highest SHAP value, i.e., 0.17, for the flowability of UHPC. In third place, cement and water have almost the same feature value for UHPC flowability. But here, the cement influences positively, showing a direct relationship, whereas the water influences negatively, depicting an inverse relationship. Afterwards, the super-plasticizer is one of the main features in UHPC, and this feature has a nearly 0.1 feature value (Figs 20 and 21). The super-plasticizer content, as a feature, is also positively influencing the flowability of UHPC. This means that increase in its content results in higher UHPC flowability. Subsequently, the sand content feature is next in position, having a feature value of around 0.08. But here, the enhancement in sand content up to optimal content increases flowability; the UHPC flowability will decrease beyond this content. This type of behavior depicts both the positive and negative influence of sand content on the flowability of UHPC. Similarly, the feature value for steel fiber length, fly ash, nano-silica, cement type, and silica-fume are next, followed by the steel fiber diameter, maximum aggregate size, quartz powder, and cement strength class. All these features show almost the same feature values below 0.05, depicting their limited influence on the flowability of UHPC.

**Fig 22:**
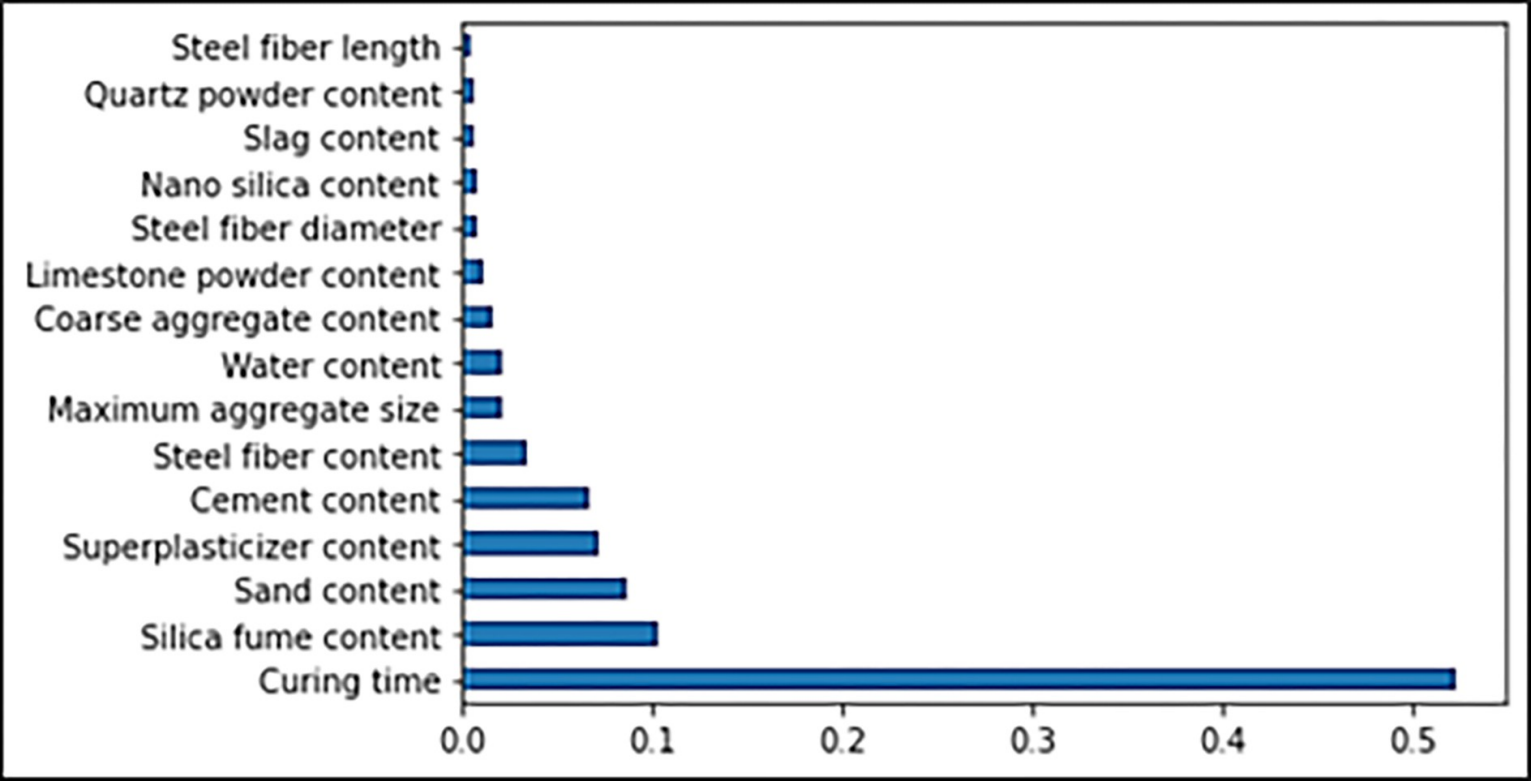
Feature importance of compressive strength.

**Fig 23:**
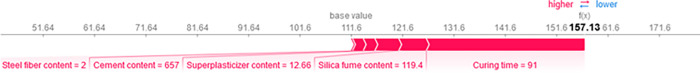
SHAP plot of compressive strength.

In the same manner, the feature and SHAP plots to show the correlation of features’ importance for compressive strength of UHPC are illustrated in Figs 22 and 23. In this plot, it is evident from Fig 22 that curing time has the maximum SHAP value, i.e., 0.52, for predicting UHPC compressive strength. It is also evident from Fig 23 that increasing the curing time would result in enhanced compressive strength of UHPC and vice versa. Although silica fume depicts the second maximum feature value, it is significantly less than the curing time. The silica-fume feature SHAP value for UHPC compressive strength is almost 0.1. The sand content feature value is in third place, followed by the cement and super-plasticizer content features, which have more or less the same SHAP values, i.e., 0.08. All the considered features for UHPC compressive strength have very little but almost the same SHAP values, i.e., less than 0.05. The nearer-to-zero SHAP value for all these features depicts these features’ minimal influence/impact on the compressive strength of UHPC. The water feature, however, is negatively influencing, meaning that with increasing water content, the compressive strength of UHPC is decreasing (Fig 22). Fig 23 also shows that the contents of silica-fume, super-plasticizer, cement and steel fiber positively influence the UHPC compressive strength, as with increasing contents, the compressive strength is enhanced and vice versa. The dataset utilized in this research is the basis of this estimation, and highly precise results may be achieved upon more data points.

## 6. Discussion and limitations

The comparison of the algorithms employed in the current study with the models applied in different studies reported in the literature is shown in Table 2. The flowability and compressive strength of UHPC are predicted by employing DT, GB and BA in this research, which is intended to have efficient and reliable outcomes compared with other literature studies. The 0.93 R^2^ value for GB results gives a comparable precision in predicting the compressive strength of UHPC. Overall, it may be observed from Table 2, as reported in literature, that GB ML models offer comparatively accurate results for compressive strength prediction of different types of concrete. It may be noted that BA, after GB, algorithms show higher precision and lesser error as compared to other algorithms and the algorithms, as reported in the literature (Table 2).

**Table 2:** Comparison of statistical checks for compressive strength prediction of different concrete types in current study with literature.

ML Approaches	Material Description	R^2^	RMSE (MPa)	MAE (MPa)	References
Decision Tree (DT)	UHPC	0.86	12.9	8.8	Current Study
Gradient Bosting (GB)	UHPC	0.93	9.2	7.0	Current Study
Bootstrap Aggregation (BA)	UHPC	0.92	9.7	7.2	Current Study
Decision Tree (DT)	Geopolymer concrete	0.88	6.2	4.1	Zou, Zheng [47]
Decision Tree (DT)	Fly ash concrete	0.83	-	-	Khan, Ahmad [48]
Adaptive Boosting (AdaBoost)	Waste marble powder Concrete (WMC)	0.91	7.9	3.9	Khan, Ahmad [49]
Gradient Bosting (GB)	Recycled coarse aggregate concrete (RCAC)	0.94	10.5	7.7	Amin, Ahmad [50]
Bootstrap Aggregation (BA)	Fly ash concrete	0.93	-	-	Khan, Ahmad [48]
Multiple Layer Perceptron Neural Network (MLPNN)	Geopolymer concrete	0.81	7.4	5.8	Amin, Khan [51]
Support Vector Machine (SVM)	Geopolymer concrete	0.78	8.1	6.7	Amin, Khan [51]

However, the researchers should also be well aware of the challenges and limitations in ML algorithms. Initially, the challenge is to select an adequate ML algorithm that is not simple for scholars in concrete engineering who are unfamiliar with ML. Several ML approaches have been employed for various concrete types, but still, no coherence has been developed. In some research studies, the superior performance of decision tree-based algorithms is reported compared to neural network-based algorithms, whereas the other researchers reported the vice versa [52]. Different machine learning algorithms have been employed by various researchers for predicting the concrete material and concrete member properties [53–56]. Though numerous benchmarking and comparison studies are available in the literature for evaluating an adequate model in the case of different concrete types, but the results are still inconsistent. This inconsistency might be due to using variable mix proportions or databases for the performance prediction of the same materials. It is also noteworthy that model’s reliability is considerably based on the adopted mix proportions/ database incorporated for evaluation, which restricts the researchers from applying the ML technique. Provided that every above-mentioned ML model has discrete pros and cons, an adequate model is selected based on the variables’ variety. The type of link between the mechanical strength of concrete and its components is a considerable aspect inducing the model selection. Accordingly, the current study determined a comparative assessment of different ML techniques and their respective efficiency to predict the UHPC flowability and compressive strength.

## 7. Conclusions

In the recent past, the employment of artificial intelligence (AI) techniques is gaining attention in the construction field for predicting concrete’s mechanical properties. The primary focus of this research is to evaluate the level of accuracy of AI approaches in predicting the flowability and compressive strength of UHPC. The input dataset comprises the contents of cement (Kg/m^3^), fly-ash (Kg/m^3^), silica-fume (Kg/m^3^), slag (Kg/m^3^), nano-silica (Kg/m^3^), quartz powder (Kg/m^3^), limestone powder (Kg/m^3^), sand (Kg/m^3^), coarse aggregates (Kg/m^3^), super-plasticizers (Kg/m^3^), steel fiber (%), polystyrene fibers (%) and water (Kg/m^3^), type, strength-class and compressive strength (MPa) of cement, maximum aggregate size (mm), and length (mm) and diameter (μm & mm) of polystyrene fiber and steel fiber. The following conclusions are made based on the conducted study.

- The Gradient Boosting approach is recommended to predict UHPC flowability accurately, as evident by the 0.92 R^2^ value. At the same time, ensembled Bootstrap aggregating and individual decision tree ML algorithms bearing 0.86 and 0.83 R^2^ values, respectively, show acceptable results for the flowability of UHPC. The higher R^2^ and lesser MAE and RMSE values are attained from k-fold cross-validation results in the GB algorithms with respect to other considered models for UHPC flowability. UHPC predicted flowability is optimized based on 20 sub-models in between 10–200 predictors. The more effective prediction for the flowability of UHPC via the ensembled DT model is observed compared to other considered models. In terms of statistical checks such as MAE and RMSE, again, the more determination coefficient and lesser error values for the flowability of UHPC in the case of GB depict its superiority among other models. Hence, Gradient Boosting is the best prediction model for the flowability of UHPC. It is revealed from the SHAP plot that the steel fiber content is positively influencing the UHPC flowability. SHAP analysis further reveals that the limestone powder content has the maximum influence on UHPC flowability prediction, followed by steel fiber, cement and water contents. The cement strength class influences the least on flowability of UHPC.
- Regarding the compressive strength of UHPC, the GB approach has shown an accurate prediction, as represented by the 0.93 R^2^ value. But the 0.92 and 0.86 R^2^ values for ensembled Bootstrap aggregating and individual DT ML models, respectively, represent the acceptable results for the compressive strength of UHPC. The optimization of estimated compressive strength for UHPC is made by using 20 sub-models ranging from 10 to 200 estimators. Here again, the ensembled GB model shows a highly precise prediction for the compressive strength of UHPC. The k-fold cross-validation method also shows the superiority of GB models over other considered models due to more R^2^ and lesser MAE and RMSE values for the compressive strength of UHPC. The same is evident from the higher coefficient of determination and lesser error values in the case of GB. Hence it can be said that GB is the best prediction algorithm for the compressive strength of UHPC. SHAP analysis reveals that the curing time feature significantly influences UHPC compressive strength prediction, followed by silica fume, sand, and super-plasticizer contents. The length of steel fiber least influences the compressive strength of UHPC. It is revealed from the SHAP plot that curing time is positively influencing the UHPC compressive strength.
- As compared to typical concrete, ultra-High-Performance Concrete (UHPC) is an advanced composite with considerably improved mechanical and durability characteristics [57–60]. The interest is rising in research and commercial utilization of UHPC. But, depending on the conducted study related to advanced algorithms, the following prospects are proposed:
- The relationship nature among raw ingredients of concrete mix design and its properties is a primary factor that impacts the ML model selection. The nonlinearity of this relationship demands the employment of ML models. However, optimizing employed models by metaheuristic algorithms is a practical approach to getting highly precise outcomes and improving processes.
- In order to employ ML approaches for the prediction of UHPC properties, users lacking in knowledge regarding ML basics, such as concrete engineers, should evaluate their employed ML-based algorithms before recommending them for practical implementation.
- Though the data sets of diverse variety are being utilized in the current study, the input variables database should be further expanded to enhance the responsiveness of the employed ML models. Additionally, enhancing quantity of data samples by extensive experimental investigations can improve the ML models’ performance in more precise prediction.
- Furthermore, enhancing the input variables of UHPC, such as the raw ingredients’ chemical composition and environmental effects (i.e., temperature and humidity), may lead towards better accuracy of ML algorithms.
- Moreover, the current work exclusively focused on predicting UHPC flowability and compressive strength, so its flexural and tensile behaviors should also be considered in future via ML prediction.
- The employment of hybrid ML algorithms may also be done to predict UHPC properties in terms of process and precision. Though it would enhance the computation time, employing these algorithms on extended datasets with adequate feature selection would offer highly precise results.
- Last, the information regarding Life Cycle Cost Analysis (LCCA) and Life Cycle Assessment (LCA) UHPC by employing AI approaches is also important to be explored before its practical implementation and large-scale production.

## List of abbreviations

AI: Artificial Intelligence
BA: Bootstrap Aggregating
DT: Decision Tree
GB: Gradient Boosting
MAE: Mean Absolute Error
ML: Machine Learning
MLPNN: Multiple Layer Perceptron Neural Network
R^2^: Determination Coefficient
RAC: Recycled Aggregate Concrete
RMSE: Root Mean Square Error
SCM: Supplementary Cementitious Materials
SHAP: SHapley Additive exPlanations
SVM: Support Vector Machine
UHPC: Ultra-High-Performance Concrete
